# Accuracy of the diagnosis of malignant hyperthermia in hospital discharge records

**DOI:** 10.1186/1471-2253-14-S1-A23

**Published:** 2014-08-18

**Authors:** Teeda Pinyavat, Henry Rosenberg, Barbara H Lang, Cynthia A Wong, Sheila Riazi, Joanne Brady, Lena Sun, Guohua Li

**Affiliations:** 1 Department of Anesthesiology, Columbia University College of Physicians and Surgeons, New York City, NY 10032, USA; 2 Department of Medical Education and Clinical Research, Saint Barnabas Medical Center, Livingston, NJ 07039, USA; 3 Department of Anesthesiology, Northwestern University Feinberg School of Medicine, Chicago, IL 60611, USA; 4 Department of Anesthesiology, Toronto General Hospital, Toronto, M5G 2C4, Canada; 5 Departments of Anesthesiology and Epidemiology, Columbia University College of Physicians and Surgeons, New York City, NY 10032, USA

## Background

In 1997, the International Classification of Diseases, 9^th^ Revision Clinical Modification (ICD-9CM) coding system introduced the code for malignant hyperthermia (MH) (995.86). The aim of the current study was to estimate the accuracy of coding for MH in hospital discharge records.

## Materials and methods

A panel of anesthesiologists expert in MH, reviewed medical records for patients with a discharge diagnosis of MH based on ICD-9 or ICD-10 codes from January 1, 2006 to December 31, 2008 at six tertiary care medical centers in North America. All cases were categorized as possible, probable, or fulminant MH, history of MH (family or personal) or other.

## Results

A total of 47 medical records were identified and reviewed by three experts. The mean age of patients was 40 years and 49% were male. A surgical procedure with general anesthesia was documented in 68% of patients. However, only 23.4% were judged to have had a possible, probable, or fulminant MH event. Dantrolene was given in 81% of MH cases. Family and personal history of MH accounted for 46.8% of cases. High fever without evidence of MH during admission accounted for 23.4%, and in 6.4% cases the reason for the code was not apparent. All patients judged to have an incident MH event survived to discharge.

## Conclusions

Medical record coding for MH typically includes both incident cases as well as a history of MH. The positive predictive value of about 70% for MH in this study are consistent with other studies of ICD-9 accuracy in the US. However, epidemiologic studies based on coded diagnosis of MH should carefully distinguish between incident cases related to anesthesia, cases unrelated to anesthesia and diagnosis based on history only.

